# Cancer-Associated Function of 2-Cys Peroxiredoxin Subtypes as a Survival Gatekeeper

**DOI:** 10.3390/antiox7110161

**Published:** 2018-11-11

**Authors:** Sang Won Kang, Sunmi Lee, Joanna H. S. Lee

**Affiliations:** 1 Department of Life Science and the Research Center for Cell Homeostasis, Ewha Womans University, Seoul 03760, Korea; shah-5@hanmail.net (S.L.); joannalee@ewhain.net (J.H.S.L.); 2 Science Bldg. C/Room 504, 52 Ewhayeodae-gil, Seodaemun-gu, Seoul 120-750, Korea

**Keywords:** peroxiredoxin, cancer, apoptosis, autophagy, chemical inhibitor

## Abstract

Cancer cells are abnormal cells that do not comply with tissue homeostasis but undergo uncontrolled proliferation. Such abnormality is driven mostly by somatic mutations on oncogenes and tumor suppressors. Cancerous mutations show intra-tumoral heterogeneity across cancer types and eventually converge into the self-activation of proliferative signaling. While transient production of intracellular reactive oxygen species (ROS) is essential for cell signaling, its persistent production is cytotoxic. Thus, cancer cells require increased levels of intracellular ROS for continuous proliferation, but overexpress cellular peroxidase enzymes, such as 2-Cys peroxiredoxins, to maintain ROS homeostasis. However, suppression of 2-Cys peroxiredoxins has also been reported in some metastatic cancers. Hence, the cancer-associated functions of 2-Cys peroxiredoxins must be illuminated in the cellular context. In this review, we describe the distinctive signaling roles of 2-Cys peroxiredoxins beyond their intrinsic ROS-scavenging role in relation to cancer cell death and survival.

## 1. Introduction

Compared with normal cells, cancer cells produce more reactive oxygen species (ROS) intracellularly, which confers their hyperproliferative capability [1,2]. However, it is a paradox that cancer cells fortify their antioxidant systems to maintain steady-state levels of ROS within the tolerable range. Major cellular antioxidant systems include the glutathione reductase—glutathione (GSH)—glutathione peroxidase (Gpx) and thioredoxin reductase—thioredoxin (Trx)—peroxiredoxin (Prx) axes, both of which are well conserved from bacteria to human [3,4]. Gpx and Prx enzymes are the type of thiol peroxidases that mainly expend the reduced nicotinamide adenine dinucleotide phosphate (NADPH) as an ultimate reducing power for eliminating H_2_O_2_. Therefore, it is understandable that the metabolism of cancer cells is biased to the pentose phosphate pathway that generates NADPH [5]. Unlike the Gpx enzyme which has a selenocysteine residue at the active site, the H_2_O_2_ reduction reaction by Prx is mediated by cysteine residues. Depending on the number of active-site cysteine residues, the Prx family is divided into the 2-Cys and 1-Cys subfamilies composed of five and one members, respectively [3]. Prx I through V, belonging to the 2-Cys Prx subfamily, are Trx-dependent thiol peroxidases and distributed throughout the cellular compartments, i.e., Prx I and II in cytosol and nuclei [6]; Prx III in mitochondria [7]; Prx IV in endoplasmic reticulum and extracellular space [8]; and Prx V in cytosol, mitochondria, and peroxisome [9]. In terms of its enzymatic mechanism, Prx V is distinct from the others due to forming an intramolecular disulfide linkage between the peroxidatic and resolving cysteine residues upon reaction with H_2_O_2_.

2-Cys Prxs also receive attention as multifunctional enzymes. Due to the unusual architecture of the dimeric structure, yeast and mammalian 2-Cys Prxs are inactivated during the reaction cycle by hyperoxidation of the peroxidatic cysteine residue into sulfinic acid [10,11]. However, it has been shown that the hyperoxidized 2-Cys Prxs are inactive for peroxidase activity but gain a new chaperonic activity by forming a high-molecular-weight oligomeric structure [12]. More recently, several interesting studies have revealed the role of cytosolic 2-Cys Prxs as a redox relay in H_2_O_2_-mediated signal transduction. Similar to the protein disulfide isomerase-like activity of Prx IV in the endoplasmic reticulum [13], the cytosolic 2-Cys Prxs primarily react with the cellular H_2_O_2_ and then transduce their disulfide to the client proteins [14]. Prx I is oxidized upon H_2_O_2_ treatment and then transduces the disulfide to apoptosis-inducing kinase (ASK1) [15]. A disulfide exchange intermediate was found between Prx II and signal transducer and activator of transcription 3 (STAT3) transcription factor [16]. Moreover, it has been shown that the disulfide formation in some cytosolic proteins requires the presence of cytosolic 2-Cys Prxs [17].

In 2005, we reviewed numerous studies with respect to the altered expression of 2-Cys Prxs in various cancer types [18]. In particular, Prx I was the most prominent subtype with increased expression in tumor tissues and later became an Nrf2 target gene [19]. The altered expression of 2-Cys Prxs is now well summarized in the Human Protein Atlas database [20]. Subsequently, recent studies including ours have reported interesting observations wherein Prx II and Prx IV are silenced by promoter methylation in various cancers [21,22,23,24,25]. The tissue-specific silencing of 2-Cys Prxs provides an important clue that 2-Cys Prx subtypes have distinct roles in tumorigenesis and cancer development. In this review, we summarize the involvement of 2-Cys Prxs in the death and survival of cancer cells and elucidate cancer treatment targeting 2-Cys Prx.

## 2. Transcriptional Regulation of 2-Cys Prxs in Cancer Cells

**Figure 1:**
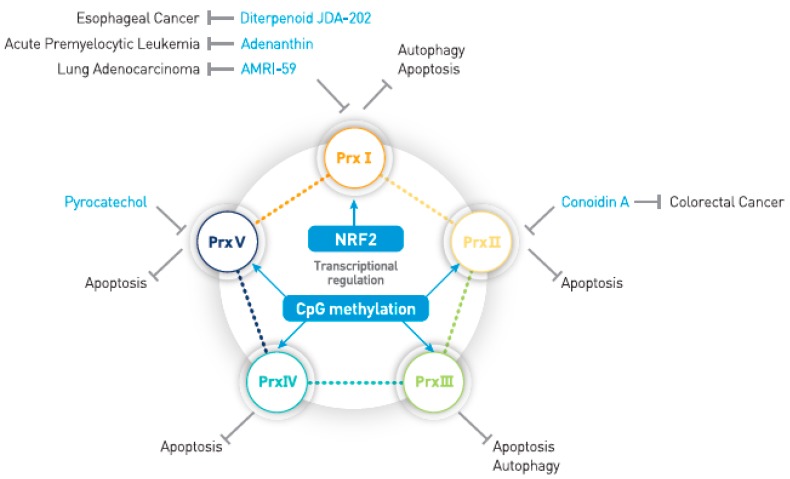
Regulation of the expression and activity of 2-Cys peroxiredoxins (Prxs). Prx I expression is mainly regulated by nuclear factor (erythroid-derived 2)-like 2 (Nrf2), whereas Prx II through V are regulated by promoter methylation. Note that hyper-methylation on the Prx I promoter has been reported in oligodendroglial tumors. To date, four compounds (conoidin A, adenanthin, AMRI59, and JDA-202) have been validated for inhibiting Prx I and/or Prx II in vitro and in vivo. As 2-Cys Prxs suppress the apoptosis of cancer cells, chemical inhibitors against 2-Cys Prxs are most likely to induce apoptosis in cancer cells. CpG: cytosine-guanine site.

Cancer cells survive various internal or external stresses by upregulating multiple survival genes, among which are antioxidant enzymes represented by 2-Cys Prxs. The master transcription factor that directs the expression of antioxidant proteins is nuclear factor E2-related factor 2 (Nrf2). Nrf2 is ubiquitously expressed but maintained at low levels under normal conditions in which its protein level is in turn controlled by the essential negative regulator Kelch-like ECH-associated protein 1 (KEAP1)/CUL3-RBX1 E3 ligase complex [26]. During oxidative stress, KEAP1 is oxidized and dissociated from Nrf2, which then translocates to the nucleus and binds to the antioxidant response element (ARE) for the transcription of target genes involved in cellular antioxidant defense [26]. Among the 2-Cys Prxs, Prx I was found to be the target gene of Nrf2 [19]. In particular, high expression of Prx I in lung adenocarcinoma cells is correlated to Nrf2 activation by somatic mutations in the Nrf2–KEAP1 pathway [27]. Besides the Nrf2-dependent transcriptional regulation, gene silencing by CpG methylation on the promoter region appears to be more common among 2-Cys Prxs (Figure 1). For example, Prx I expression is silenced in oligodendroglial tumors by promoter methylation, which confers radio- and chemo-sensitivity [28]. Silencing of Prx II by promoter methylation was found in melanoma, stomach cancer, acute myeloid leukemia, and Hodgkin lymphoma cells [21,22,23,24]. In melanoma, silencing of Prx II expression promotes Src and ERK activation, which increases migratory activity and, hence, metastasis [29]. Prx IV was shown to be silenced in acute promyelocytic leukemia, but not in acute myeloid leukemia [25]. On the other hand, the post-transcriptional regulation of 2-Cys Prxs by microRNA has frequently been observed depending on cancer types. As such, an elevated level of miR510 is seen to negatively regulate Prx I expression in breast cancer [30]. Prx II expression is downregulated by miR-200b in some colorectal cancer cell types, CaCo_2_ and SW480, which enhances metastasis and drug resistance [31]. Prx II expression is also downregulated by miR153-3p and miR205-5p in neuroblastoma cells [32]. While Prx III expression is down-regulated by miR23b-3p and miR26a-5p in prostate cancer and acute myeloid leukemia, it is upregulated in medulloblastoma due to the lowered miR383 level [33,34,35]. This overview contradicts our preconception that Prx is one of the ROS-scavenging antioxidant enzymes usually overexpressed in tumors. Such complex transcriptional regulation of 2-Cys Prxs in cancers implicates the potential role of Prxs as a key regulator of redox signaling in cancer cell growth and survival.

## 3. Regulation of Cancer Cell Death by 2-Cys Prxs

Differential expression of 2-Cys Prxs among various cancer types reflects that the cellular redox state is an adaptive response of cancer cells against external or internal oxidative stress leading to cell death. Mammalian cells undergo morphologically and biochemically distinct types of programmed cell death, such as apoptosis and necroptosis. Apoptosis is caspase-dependent programmed cell death and is induced by death receptor ligands, such as TNF-α and FasL [36]. One of the major apoptosis pathways is the intrinsic process involving the dissipation of mitochondria membrane permeability transition (mPT, ΔΨ) via Bax/Bak macropore, which in turn triggers robust production of ROS. Since the caspases executing apoptosis are susceptible to H_2_O_2_-dependent inactivation [37,38], the role of ROS in apoptosis may be context dependent. By contrast, necroptosis is caspase-independent and receptor-interacting protein kinase-3 (RIPK3)-dependent programmed necrotic death induced by TNF-α [39]. In necroptosis, mitochondrial outer membrane permeabilization (MOMP) is involved in robust ROS production [40]. Hence, it is worth looking into the role of 2-Cys Prxs in cell death signaling (Figure 1). The first evidence of the role of 2-Cys Prx in relation to cell survival or death was found in 1997 by Shau et al., who discovered that Prx II, also referred to as natural killer enhancing factor (NKEF)-B, protects human endothelial cells against oxidative stress and chemotherapeutic agents [41]. A subsequent work reported that Prx I and Prx II overexpression inhibit H_2_O_2_-induced apoptosis in rat thyroid cells [42]. The first cancer cell study focused on head and neck cancer cells wherein the knockdown of Prx II increased the sensitivity of cancer cells to radiation-induced deaths [43]. Numerous studies have indicated that 2-Cys Prxs are overexpressed in various human tumors compared to normal tissues [44,45,46,47,48]. Nonetheless, the molecular mechanism by which 2-Cys Prxs regulate cancer cell apoptosis has been rarely studied. The first detailed study was conducted using the RNAi system and showed that Prx III inhibits the mitochondrial mPT and cytochrome c release in HeLa cervical cancer cells treated with tumor necrosis factor (TNF)-α and staurosporine [7]. Later, it was determined that Prx III is primarily oxidized during the early stage of Fas-induced apoptosis in Jurkat T cells [49]. Two independent groups have shown that Prx V plays an anti-apoptotic role in HeLa cervical cancer and U1810 lung adenocarcinoma cells [50,51]. We have shown that Prx II regulates TNF-α- and TNF-related apoptosis-inducing ligand (TRAIL)-induced apoptosis via the FADD–Caspase 8–Caspase-3 pathway [52]. Contradictory studies exist on the role of Prx I in apoptosis. For example, one report showed that Prx I catalyzed ASK1 oxidation and activation by forming a mixed disulfide intermediate between two proteins [15], claiming a novel role of Prx I as the peroxide receptor and transducer in the H_2_O_2_-induced apoptosis. However, another study showed that Prx I is overexpressed in oral precancerous lesions and required for a high malignant growth rate by suppressing ASK1/p38 activation [53]. In general, most of the previous studies implicate a protective role of 2-Cys Prxs against apoptosis in cancer cells.

## 4. H_2_O_2_-Dependent Autophagic Control in Cancer Cells

Since autophagy regulates the turnover of long-lived proteins and subcellular organelles under basal conditions [54], its impairment may cause chronic inflammation and tumor initiation. Enigmatically, autophagy is a highly activated stress adaptation response in established cancer cells experiencing oxidative and metabolic stresses [55,56]. Hence, ROS are thought to be one of the key autophagy inducers [57]. For instance, mitophagy is the autophagic process eliminating damaged mitochondria that actively produce ROS in cancer cells. A decade ago, it was already shown that ATG4, which is a cysteine protease that mediates autophagosome formation by regulating microtubule-associated protein 1A/1B-light chain 3 (LC3, Atg8-homologue) states, is activated by cysteine oxidation by H_2_O_2_ [58]. A later study revealed that H_2_O_2_-dependent oxidation mediates the formation of a disulfide linkage between Cys338 and Cys 394 on yeast ATG4, which was reduced by thioredoxin [59]. These studies provided evidence of ATG4 being the molecular target linking H_2_O_2_ to autophagy. However, a few studies have been conducted to understand the role of 2-Cys Prxs in autophagy. For example, Prx II reduces pulmonary inflammatory vasculopathy in mice by down-regulating autophagy [60]. Prx I is shown to control cholesterol homeostasis in macrophages by lipophagy, an autophagic degradation of intracellular lipid droplets, and its absence thus exacerbates atherosclerotic pathogenesis [61]. Prx III was shown to be highly expressed in benign prostatic hyperplasia tissues and inhibit autophagy [62]. Therefore, the role of 2-Cys Prx in autophagy control remains to be investigated in the context of cancer biology.

## 5. Targeting 2-Cys Prxs for Cancer Treatment

**Figure 2:**
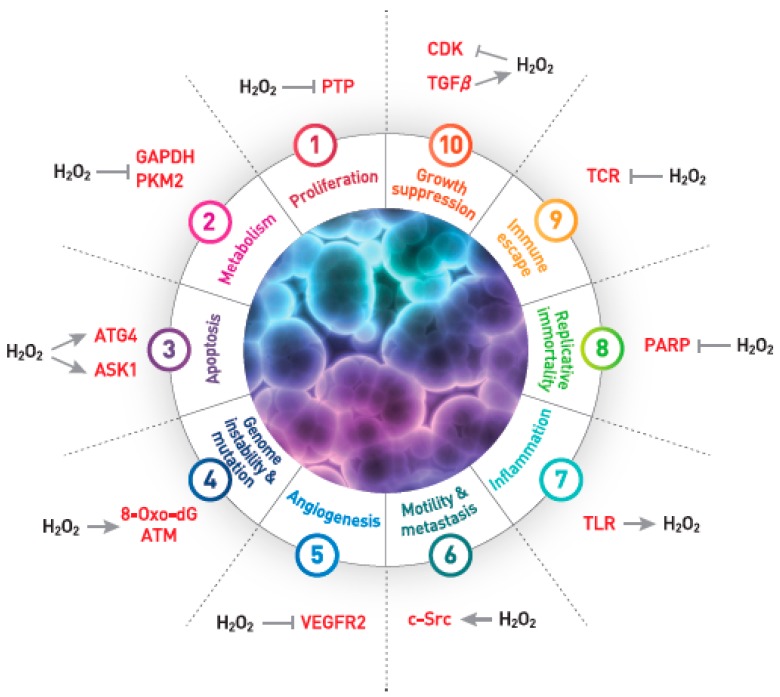
Involvement of H_2_O_2_ in the hallmarks of cancer. Key signaling molecules activated (→) or inhibited (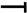) by H_2_O_2_ are depicted in relation to the hallmarks of cancer. PTP, protein tyrosine phosphatase; GAPDH, glyceraldehyde-3-phosphate dehydrogenase; PKM2, pyruvate kinase M2; ATG4, autophagy-related 4; ASK1, apoptosis signal kinase 1; 8-Oxo-dG, 8-oxo-2′-deoxyguanosine; ATM, ataxia telangiectasia mutated; VEGFR2, vascular endothelial growth factor 2; c-Src, TLR, toll-like receptor; PARP, poly(ADP-ribose) polymerase; TCR, T cell receptor; CDK, cyclin-dependent kinase; TGF, transforming growth factor.

It is undeniable that altering the intracellular ROS level influences the biological activity of cancer cells. The following evidence from past decades highlights that ROS are the key component in the hallmark of cancer as described by Hanahan and Weinberg [63] (Figure 2): (1) ROS are the second messenger in cell growth signaling [64]; (2) ROS play a critical role in cancer metabolism [65]; (3) ROS are involved in the apoptosis of cancer cells as described above; (4) ROS induce genome instability by somatic DNA mutation [66]; (5) ROS can interfere with angiogenesis via oxidative inactivation of VEGFR2 [67]; (6) ROS promote the motility of mammalian cells including cancer cells [29,68]; (7) ROS are produced by immune cells in inflammatory sites [69]; and (8) ROS are involved in the activation of immune cells combating against cancer cells [70]. Despite tremendous efforts to develop oxidants and antioxidants as anticancer drugs, the FDA-approved anticancer drugs targeting ROS are still unavailable in the market [1]. We therefore posit that the reason for the lack of success is that, as aforementioned, the expression level of 2-Cys Prx varies depending on the tissue and cell-type origins of cancer cells. In other words, a general antioxidant therapy does not work for cancer treatment due to tumor heterogeneity. Therefore, targeting a distinct type of 2-Cys Prx with respect to a particular type of cancer may be a promising anticancer strategy.

To date, a few compounds have been identified as small molecule inhibitors targeting 2-Cys Prx (Figure 1). The first 2-Cys Prx inhibitor was discovered in the course of target identification for a quinoxalin compound, conoidin A, which was shown to inhibit host cell invasion by the human pathogen *Toxoplasma gondii* [71,72]. Although conoidin A was initially revealed to inhibit the peroxidase activity of the parasitic Prx II, we have recently shown that it also inhibits both human Prx I and Prx II [73]. Interestingly, conoidin A selectively killed colorectal cancer cells with an adenomatous polyposis coli (APC) mutation in vitro and in vivo. The second inhibitor compound targeting 2-Cys Prx is adenanthin, a natural compound isolated from plants [74]. Although adenanthin has been shown to inhibit Prx I and II, it was later claimed to be a selective inhibitor of thioredoxin reductase [75]. Since Soethoudt et al. showed that adenanthin treatment also reduces glutathione levels in erythrocytes and Jurkat T cells, it is conceivable that adenanthin is to some extent a thiol-reacting compound. In any case, adenanthin repressed tumor growth in leukemic mice by inducing CCAAT/enhancer binding protein-β (C/EBPβ) activation. Recently, two more Prx I-selective inhibitors were reported: (1) AMRI-59 was specifically shown to inhibit Prx I and consequently led to the apoptosis of A549 lung adenocarcinoma in vitro and in a xenografted tumor [76]; and (2) a natural diterpenoid, JDA-202, was shown to inhibit Prx I activity in vitro and promote H_2_O_2_-induced apoptotic death in esophageal cancer cells, in which the Prx I level is upregulated [77]. Considering that genetic ablation of Prx I caused severe malignant cancer in aged mice [78] and promoted K-Ras-driven lung tumorigenesis in mice [79], cancer treatment targeting Prx I may need careful attention in its approach. In addition, 4-substituted pyrocatechol was proposed to be a potential inhibitor of Prx V isoform by a computational simulation [80] which must be validated. Conversely, we have shown that the compounds mimicking 2-Cys Prx activity inhibit melanoma metastasis [29]. Only one type of 2-Cys Prx mimicry was discovered among the family of fungal secondary metabolites named epidithiopiperazine (ETP) [81]. These activity mimicry compounds were shown to be effective in cancer cells harboring silenced Prx II expression [21]. Hence, choosing the appropriate chemical tools to control 2-Cys Prx activity between inhibition and mimicry is dependent on the context of cancer biology.

## 6. Conclusions and Remarks

ROS determine the fate of cancer cells in terms of proliferation, migration, and death. As we summarize in this review, specific targeting of 2-Cys Prxs may be a more effective approach than the precarious application of non-specific pro- or anti-oxidant compounds for cancer therapy via controlling cellular ROS function.

## References

[1] Trachootham D., Alexandre J., Huang P. (2009). Targeting cancer cells by ROS-mediated mechanisms: A radical therapeutic approach?. Nat. Rev. Drug Discov..

[2] Cerutti P.A. (1994). Oxy-radicals and cancer. Lancet.

[3] Rhee S.G., Chae H.Z., Kim K. (2005). Peroxiredoxins: A historical overview and speculative preview of novel mechanisms and emerging concepts in cell signaling. Free Radic. Biol. Med..

[4] Lubos E., Loscalzo J., Handy D.E. (2011). Glutathione peroxidase-1 in health and disease: From molecular mechanisms to therapeutic opportunities. Antioxid. Redox Signal..

[5] Pavlova N.N., Thompson C.B. (2016). The Emerging Hallmarks of Cancer Metabolism. Cell Metab..

[6] Lee K.W., Lee D.J., Lee J.Y., Kang D.H., Kwon J., Kang S.W. (2011). Peroxiredoxin II restrains DNA damage-induced death in cancer cells by positively regulating JNK-dependent DNA repair. J. Biol. Chem..

[7] Chang T.S., Cho C.S., Park S., Yu S., Kang S.W., Rhee S.G. (2004). Peroxiredoxin III, a mitochondrion-specific peroxidase, regulates apoptotic signaling by mitochondria. J. Biol. Chem..

[8] Tavender T.J., Sheppard A.M., Bulleid N.J. (2008). Peroxiredoxin IV is an endoplasmic reticulum-localized enzyme forming oligomeric complexes in human cells. Biochem. J..

[9] Seo M.S., Kang S.W., Kim K., Baines I.C., Lee T.H., Rhee S.G. (2000). Identification of a new type of mammalian peroxiredoxin that forms an intramolecular disulfide as a reaction intermediate. J. Biol. Chem..

[10] Wood Z.A., Poole L.B., Karplus P.A. (2003). Peroxiredoxin evolution and the regulation of hydrogen peroxide signaling. Science.

[11] Yang K.S., Kang S.W., Woo H.A., Hwang S.C., Chae H.Z., Kim K., Rhee S.G. (2002). Inactivation of human peroxiredoxin I during catalysis as the result of the oxidation of the catalytic site cysteine to cysteine-sulfinic acid. J. Biol. Chem..

[12] Jang H.H., Lee K.O., Chi Y.H., Jung B.G., Park S.K., Park J.H., Lee J.R., Lee S.S., Moon J.C., Yun J.W. (2004). Two enzymes in one: Two yeast peroxiredoxins display oxidative stress-dependent switching from a peroxidase to a molecular chaperone function. Cell.

[13] Zito E., Melo E.P., Yang Y., Wahlander A., Neubert T.A., Ron D. (2010). Oxidative protein folding by an endoplasmic reticulum-localized peroxiredoxin. Mol. Cell.

[14] Winterbourn C.C., Hampton M.B. (2015). Redox biology: Signaling via a peroxiredoxin sensor. Nat. Chem. Biol..

[15] Jarvis R.M., Hughes S.M., Ledgerwood E.C. (2012). Peroxiredoxin 1 functions as a signal peroxidase to receive, transduce, and transmit peroxide signals in mammalian cells. Free Radic. Biol. Med..

[16] Sobotta M.C., Liou W., Stocker S., Talwar D., Oehler M., Ruppert T., Scharf A.N., Dick T.P. (2015). Peroxiredoxin-2 and STAT3 form a redox relay for H_2_O_2_ signaling. Nat. Chem. Biol..

[17] Stocker S., Maurer M., Ruppert T., Dick T.P. (2018). A role for 2-Cys peroxiredoxins in facilitating cytosolic protein thiol oxidation. Nat. Chem. Biol..

[18] Kang S.W., Rhee S.G., Chang T.S., Jeong W., Choi M.H. (2005). 2-Cys peroxiredoxin function in intracellular signal transduction: Therapeutic implications. Trends Mol. Med..

[19] Kim Y.J., Ahn J.Y., Liang P., Ip C., Zhang Y., Park Y.M. (2007). Human prx1 gene is a target of Nrf2 and is up-regulated by hypoxia/reoxygenation: Implication to tumor biology. Cancer Res..

[20] Uhlen M., Oksvold P., Fagerberg L., Lundberg E., Jonasson K., Forsberg M., Zwahlen M., Kampf C., Wester K., Hober S. (2010). Towards a knowledge-based Human Protein Atlas. Nat. Biotechnol..

[21] Hong S.H., Min C., Jun Y., Lee D.J., Kim S.H., Park J.H., Cheong J.H., Park Y.J., Kim S.Y., Lee S. (2018). Silencing of peroxiredoxin II by promoter methylation is necessary for the survival and migration of gastric cancer cells. Exp. Mol. Med..

[22] Schneider M., Szaumkessel M., Richter J., Ammerpohl O., Hansmann M.L., Kuppers R., Siebert R., Giefing M. (2014). The PRDX2 gene is transcriptionally silenced and de novo methylated in Hodgkin and Reed-Sternberg cells of classical Hodgkin lymphoma. Blood.

[23] Agrawal-Singh S., Isken F., Agelopoulos K., Klein H.U., Thoennissen N.H., Koehler G., Hascher A., Baumer N., Berdel W.E., Thiede C. (2012). Genome-wide analysis of histone H3 acetylation patterns in AML identifies PRDX2 as an epigenetically silenced tumor suppressor gene. Blood.

[24] Furuta J., Nobeyama Y., Umebayashi Y., Otsuka F., Kikuchi K., Ushijima T. (2006). Silencing of Peroxiredoxin 2 and aberrant methylation of 33 CpG islands in putative promoter regions in human malignant melanomas. Cancer Res..

[25] Palande K.K., Beekman R., van der Meeren L.E., Beverloo H.B., Valk P.J., Touw I.P. (2011). The antioxidant protein peroxiredoxin 4 is epigenetically down regulated in acute promyelocytic leukemia. PLoS ONE.

[26] Rojo de la Vega M., Chapman E., Zhang D.D. (2018). NRF2 and the Hallmarks of Cancer. Cancer Cell.

[27] Jung B.J., Yoo H.S., Shin S., Park Y.J., Jeon S.M. (2018). Dysregulation of NRF2 in Cancer: From Molecular Mechanisms to Therapeutic Opportunities. Biomol. Ther..

[28] Dittmann L.M., Danner A., Gronych J., Wolter M., Stuhler K., Grzendowski M., Becker N., Bageritz J., Goidts V., Toedt G. (2012). Downregulation of PRDX1 by promoter hypermethylation is frequent in 1p/19q-deleted oligodendroglial tumours and increases radio- and chemosensitivity of Hs683 glioma cells in vitro. Oncogene.

[29] Lee D.J., Kang D.H., Choi M., Choi Y.J., Lee J.Y., Park J.H., Park Y.J., Lee K.W., Kang S.W. (2013). Peroxiredoxin-2 represses melanoma metastasis by increasing E-Cadherin/beta-Catenin complexes in adherens junctions. Cancer Res..

[30] Guo Q.J., Mills J.N., Bandurraga S.G., Nogueira L.M., Mason N.J., Camp E.R., Larue A.C., Turner D.P., Findlay V.J. (2013). MicroRNA-510 promotes cell and tumor growth by targeting peroxiredoxin1 in breast cancer. Breast Cancer Res. BCR.

[31] Lv Z., Wei J., You W., Wang R., Shang J., Xiong Y., Yang H., Yang X., Fu Z. (2017). Disruption of the c-Myc/miR-200b-3p/PRDX2 regulatory loop enhances tumor metastasis and chemotherapeutic resistance in colorectal cancer. J. Transl. Med..

[32] Patil K.S., Basak I., Pal R., Ho H.P., Alves G., Chang E.J., Larsen J.P., Moller S.G. (2015). A Proteomics Approach to Investigate miR-153-3p and miR-205-5p Targets in Neuroblastoma Cells. PLoS ONE.

[33] Jiang W., Min J., Sui X., Qian Y., Liu Y., Liu Z., Zhou H., Li X., Gong Y. (2015). MicroRNA-26a-5p and microRNA-23b-3p up-regulate peroxiredoxin III in acute myeloid leukemia. Leuk. Lymphoma.

[34] He H.C., Zhu J.G., Chen X.B., Chen S.M., Han Z.D., Dai Q.S., Ling X.H., Fu X., Lin Z.Y., Deng Y.H. (2012). MicroRNA-23b downregulates peroxiredoxin III in human prostate cancer. FEBS Lett..

[35] Li K.K., Pang J.C., Lau K.M., Zhou L., Mao Y., Wang Y., Poon W.S., Ng H.K. (2013). MiR-383 is downregulated in medulloblastoma and targets peroxiredoxin 3 (PRDX3). Brain Pathol..

[36] Taylor R.C., Cullen S.P., Martin S.J. (2008). Apoptosis: Controlled demolition at the cellular level. Nat. Rev. Mol. Cell Biol..

[37] Lee Y.J., Shacter E. (2000). Hydrogen peroxide inhibits activation, not activity, of cellular caspase-3 in vivo. Free Radic. Biol. Med..

[38] Baker A., Santos B.D., Powis G. (2000). Redox control of caspase-3 activity by thioredoxin and other reduced proteins. Biochem. Biophys. Res. Commun..

[39] Vanden Berghe T., Linkermann A., Jouan-Lanhouet S., Walczak H., Vandenabeele P. (2014). Regulated necrosis: The expanding network of non-apoptotic cell death pathways. Nat. Rev. Mol. Cell Biol..

[40] Vandenabeele P., Galluzzi L., Vanden Berghe T., Kroemer G. (2010). Molecular mechanisms of necroptosis: An ordered cellular explosion. Nat. Rev. Mol. Cell Biol..

[41] Shau H., Kim A.T., Hedrick C.C., Lusis A.J., Tompkins C., Finney R., Leung D.W., Paglia D.E. (1997). Endogenous natural killer enhancing factor-B increases cellular resistance to oxidative stresses. Free Radic. Biol. Med..

[42] Kim H., Lee T.H., Park E.S., Suh J.M., Park S.J., Chung H.K., Kwon O.Y., Kim Y.K., Ro H.K., Shong M. (2000). Role of peroxiredoxins in regulating intracellular hydrogen peroxide and hydrogen peroxide-induced apoptosis in thyroid cells. J. Biol. Chem..

[43] Park S.H., Chung Y.M., Lee Y.S., Kim H.J., Kim J.S., Chae H.Z., Yoo Y.D. (2000). Antisense of human peroxiredoxin II enhances radiation-induced cell death. Clin. Cancer Res..

[44] Kinnula V.L., Lehtonen S., Sormunen R., Kaarteenaho-Wiik R., Kang S.W., Rhee S.G., Soini Y. (2002). Overexpression of peroxiredoxins I, II, III, V, and VI in malignant mesothelioma. J Pathol.

[45] Noh D.Y., Ahn S.J., Lee R.A., Kim S.W., Park I.A., Chae H.Z. (2001). Overexpression of peroxiredoxin in human breast cancer. Anticancer. Res.

[46] Chang J.W., Jeon H.B., Lee J.H., Yoo J.S., Chun J.S., Kim J.H., Yoo Y.J. (2001). Augmented expression of peroxiredoxin I in lung cancer. Biochem. Biophys. Res. Commun..

[47] Yanagawa T., Iwasa S., Ishii T., Tabuchi K., Yusa H., Onizawa K., Omura K., Harada H., Suzuki H., Yoshida H. (2000). Peroxiredoxin I expression in oral cancer: A potential new tumor marker. Cancer Lett..

[48] Yanagawa T., Ishikawa T., Ishii T., Tabuchi K., Iwasa S., Bannai S., Omura K., Suzuki H., Yoshida H. (1999). Peroxiredoxin I expression in human thyroid tumors. Cancer Lett..

[49] Cox A.G., Pullar J.M., Hughes G., Ledgerwood E.C., Hampton M.B. (2008). Oxidation of mitochondrial peroxiredoxin 3 during the initiation of receptor-mediated apoptosis. Free Radic. Biol. Med..

[50] Kropotov A., Gogvadze V., Shupliakov O., Tomilin N., Serikov V.B., Tomilin N.V., Zhivotovsky B. (2006). Peroxiredoxin V is essential for protection against apoptosis in human lung carcinoma cells. Exp. Cell Res..

[51] Zhou Y., Kok K.H., Chun A.C., Wong C.M., Wu H.W., Lin M.C., Fung P.C., Kung H., Jin D.Y. (2000). Mouse peroxiredoxin V is a thioredoxin peroxidase that inhibits p53-induced apoptosis. Biochem. Biophys. Res. Commun..

[52] Lee J.Y., Jung H.J., Song I.S., Williams M.S., Choi C., Rhee S.G., Kim J., Kang S.W. (2009). Protective role of cytosolic 2-cys peroxiredoxin in the TNF-alpha-induced apoptotic death of human cancer cells. Free Radic. Biol. Med..

[53] Zhang J., Jing X., Niu W., Zhang M., Ge L., Miao C., Tang X. (2016). Peroxiredoxin 1 has an anti-apoptotic role via apoptosis signal-regulating kinase 1 and p38 activation in mouse models with oral precancerous lesions. Oncol. Lett..

[54] Mizushima N., Komatsu M. (2011). Autophagy: Renovation of cells and tissues. Cell.

[55] Zhong Z., Sanchez-Lopez E., Karin M. (2016). Autophagy, Inflammation, and Immunity: A Troika Governing Cancer and Its Treatment. Cell.

[56] Guo J.Y., Xia B., White E. (2013). Autophagy-mediated tumor promotion. Cell.

[57] Scherz-Shouval R., Elazar Z. (2011). Regulation of autophagy by ROS: Physiology and pathology. Trends Biochem. Sci..

[58] Scherz-Shouval R., Shvets E., Fass E., Shorer H., Gil L., Elazar Z. (2007). Reactive oxygen species are essential for autophagy and specifically regulate the activity of Atg4. EMBO J..

[59] Perez-Perez M.E., Zaffagnini M., Marchand C.H., Crespo J.L., Lemaire S.D. (2014). The yeast autophagy protease Atg4 is regulated by thioredoxin. Autophagy.

[60] Federti E., Matte A., Ghigo A., Andolfo I., James C., Siciliano A., Leboeuf C., Janin A., Manna F., Choi S.Y. (2017). Peroxiredoxin-2 plays a pivotal role as multimodal cytoprotector in the early phase of pulmonary hypertension. Free Radic. Biol. Med..

[61] Jeong S.J., Kim S., Park J.G., Jung I.H., Lee M.N., Jeon S., Kweon H.Y., Yu D.Y., Lee S.H., Jang Y. (2018). Prdx1 (peroxiredoxin 1) deficiency reduces cholesterol efflux via impaired macrophage lipophagic flux. Autophagy.

[62] Jiang M.Y., Han Z.D., Li W., Yue F., Ye J., Li B., Cai Z., Lu J.M., Dong W., Jiang X. (2017). Mitochondrion-associated protein peroxiredoxin 3 promotes benign prostatic hyperplasia through autophagy suppression and pyroptosis activation. Oncotarget.

[63] Hanahan D., Weinberg R.A. (2011). Hallmarks of cancer: The next generation. Cell.

[64] Rhee S.G. (2006). Cell signaling. H_2_O_2_, a necessary evil for cell signaling. Science.

[65] Kang S.W., Lee S., Lee E.K. (2015). ROS and energy metabolism in cancer cells: Alliance for fast growth. Arch. Pharm. Res..

[66] David S.S., O’Shea V.L., Kundu S. (2007). Base-excision repair of oxidative DNA damage. Nature.

[67] Kang D.H., Lee D.J., Lee K.W., Park Y.S., Lee J.Y., Lee S.H., Koh Y.J., Koh G.Y., Choi C., Yu D.Y. (2011). Peroxiredoxin II is an essential antioxidant enzyme that prevents the oxidative inactivation of VEGF receptor-2 in vascular endothelial cells. Mol. Cell.

[68] Ushio-Fukai M. (2006). Localizing NADPH oxidase-derived ROS. Sci. STKE.

[69] Schieber M., Chandel N.S. (2014). ROS function in redox signaling and oxidative stress. Curr. Biol..

[70] Lu T., Gabrilovich D.I. (2012). Molecular pathways: Tumor-infiltrating myeloid cells and reactive oxygen species in regulation of tumor microenvironment. Clin. Cancer Res..

[71] Haraldsen J.D., Liu G., Botting C.H., Walton J.G., Storm J., Phalen T.J., Kwok L.Y., Soldati-Favre D., Heintz N.H., Muller S. (2009). Identification of Conoidin a as a Covalent Inhibitor of Peroxiredoxin Ii. Org. Biomol. Chem..

[72] Carey K.L., Westwood N.J., Mitchison T.J., Ward G.E. (2004). A small-molecule approach to studying invasive mechanisms of Toxoplasma gondii. Proc. Natl. Acad. Sci. USA.

[73] Kang D.H., Lee D.J., Lee S., Lee S.Y., Jun Y., Kim Y., Kim Y., Lee J.S., Lee D.K., Lee S. (2017). Interaction of tankyrase and peroxiredoxin II is indispensable for the survival of colorectal cancer cells. Nat. Commun..

[74] Liu C.X., Yin Q.Q., Zhou H.C., Wu Y.L., Pu J.X., Xia L., Liu W., Huang X., Jiang T., Wu M.X. (2012). Adenanthin targets peroxiredoxin I and II to induce differentiation of leukemic cells. Nat. Chem. Biol..

[75] Soethoudt M., Peskin A.V., Dickerhof N., Paton L.N., Pace P.E., Winterbourn C.C. (2014). Interaction of adenanthin with glutathione and thiol enzymes: Selectivity for thioredoxin reductase and inhibition of peroxiredoxin recycling. Free Radic. Biol. Med..

[76] Yang Y.J., Baek J.Y., Goo J., Shin Y., Park J.K., Jang J.Y., Wang S.B., Jeong W., Lee H.J., Um H.D. (2015). Effective Killing of Cancer Cells Through ROS-Mediated Mechanisms by AMRI-59 Targeting Peroxiredoxin I. Antioxid. Redox Signal..

[77] Shi X.J., Ding L., Zhou W., Ji Y., Wang J., Wang H., Ma Y., Jiang G., Tang K., Ke Y. (2017). Pro-Apoptotic Effects of JDA-202, a Novel Natural Diterpenoid, on Esophageal Cancer Through Targeting Peroxiredoxin I. Antioxid. Redox Signal..

[78] Neumann C.A., Krause D.S., Carman C.V., Das S., Dubey D.P., Abraham J.L., Bronson R.T., Fujiwara Y., Orkin S.H., Van Etten R.A. (2003). Essential role for the peroxiredoxin Prdx1 in erythrocyte antioxidant defence and tumour suppression. Nature.

[79] Park Y.H., Kim S.U., Lee B.K., Kim H.S., Song I.S., Shin H.J., Han Y.H., Chang K.T., Kim J.M., Lee D.S. (2013). Prx I suppresses K-ras-driven lung tumorigenesis by opposing redox-sensitive ERK/cyclin D1 pathway. Antioxid. Redox Signal..

[80] Chow M.L., Troussicot L., Martin M., Doumeche B., Guilliere F., Lancelin J.M. (2016). Predicting and Understanding the Enzymatic Inhibition of Human Peroxiredoxin 5 by 4-Substituted Pyrocatechols by Combining Funnel Metadynamics, Solution NMR, and Steady-State Kinetics. Biochemistry.

[81] Kang D.H., Lee D.J., Kim J., Lee J.Y., Kim H.W., Kwon K., Taylor W.R., Jo H., Kang S.W. (2013). Vascular injury involves the overoxidation of peroxiredoxin type II and is recovered by the peroxiredoxin activity mimetic that induces reendothelialization. Circulation.

